# Context-Aware Superpixel and Bilateral Entropy—Image Coherence Induces Less Entropy

**DOI:** 10.3390/e22010020

**Published:** 2019-12-23

**Authors:** Feihong Liu, Xiao Zhang, Hongyu Wang, Jun Feng

**Affiliations:** 1 School of Information Science and Technology, Northwest University, Xi’an 710027, China; 201531439@stumail.nwu.edu.cn; 2 School of Computer Science and Technology, Xi’an University of Posts and Telecommunications, Xi’an 710121, China; hywang@xupt.edu.cn; 3 Shaanxi Key Laboratory of Network Data Analysis and Intelligent Processing, Xi’an University of Posts and Telecommunications, Xi’an 710121, China; 4 State-Province Joint Engineering and Research Center of Advanced Networking and Intelligent Information Services, School of Information Science and Technology, Northwest University, Xi’an 710127, China

**Keywords:** computer vision, superpixel, image representation, image entropy, minimum entropy principle, Gestalt grouping rules

## Abstract

Superpixel clustering is one of the most popular computer vision techniques that aggregates coherent pixels into perceptually meaningful groups, taking inspiration from Gestalt grouping rules. However, due to brain complexity, the underlying mechanisms of such perceptual rules are unclear. Thus, conventional superpixel methods do not completely follow them and merely generate a flat image partition rather than hierarchical ones like a human does. In addition, those methods need to initialize the total number of superpixels, which may not suit diverse images. In this paper, we first propose context-aware superpixel (CASP) that follows both Gestalt grouping rules and the top-down hierarchical principle. Thus, CASP enables to adapt the total number of superpixels to specific images automatically. Next, we propose bilateral entropy, with two aspects conditional intensity entropy and spatial occupation entropy, to evaluate the encoding efficiency of image coherence. Extensive experiments demonstrate CASP achieves better superpixel segmentation performance and less entropy than baseline methods. More than that, using Pearson’s correlation coefficient, a collection of data with a total of 120 samples demonstrates a strong correlation between local image coherence and superpixel segmentation performance. Our results inversely support the reliability of above-mentioned perceptual rules, and eventually, we suggest designing novel entropy criteria to test the encoding efficiency of more complex patterns.

## 1. Introduction

Superpixels are groups of coherent pixels. Instead of the huge amount of pixels in the uniform grid, only several superpixels in irregular shape are the elementary unit of an image, which serve in a number of subsequent computer vision methods. At the very beginning, superpixel designers take inspiration from Gestalt grouping rules [1]. Although the correlation between such perceptual rules and visual efficiency is not well interpreted yet, Gestalt researchers, based on behavioral evidence, hypothesized the minimum principle that illustrates the efficiency of visual processes [2]. On the other hand, the employment of superpixels has greatly reduced image redundancy and has significantly improved the processing efficiency of a number of computer vision methods in practice, including object segmentation [3,4,5], recognition [6], location [7] and tracking [8], and so forth.

While the underlying mechanisms of visual efficiency are not figured out yet, vision researchers continuously discover mechanisms supporting that stimuli coherence depicted by Gestalt grouping rules accelerates vision formation, even at the very early stages of perception (i.e., in the visual working memory) [9,10,11]. However, since the human brain is extremely complex, it is impossible to evaluate the efficiency of transmitting, encoding, or processing visual information in vivo so far. Only a few pioneer researchers attempt to quantitatively figure out that question in virtue of information theory [12,13]. They hypothesized that the human visual system transmits visual information with the utmost economy either with respect to time or bandwidth.

As an alternative of the brain, an increasing number of computer vision systems serve as experimental platforms in testing research hypotheses [14,15,16]. Those artificial vision systems even excel humans on a number of practical tasks, owing to the advance of computer vision techniques; and they can be a reliable tool assisting humans in routine tasks [17,18,19,20]. Therefore, a superpixel method that completely follows perceptual rules can be exploited to test the Gestalt minimum hypothesis. Nevertheless, there are limitations on conventional superpixel algorithms and the image entropy.

Firstly, conventional superpixel algorithms do not completely follow Gestalt grouping rules. Normalized cuts is an Eigen-based method [1], which is very time-consuming. Next, Achanta et al. (2012) proposed the simple linear iterative clustering (SLIC) which iteratively aggregates pixels based on the K-means clustering in a 5D Euclidean space [21]. Because SLIC can only utilize local image characteristics, which is less effective, Li and Chen proposed linear spectral clustering (LSC) which can capture perceptually important global features [22]. Nevertheless, these methods only generate the flat image partition rather than hierarchical image structures like a human does. Besides, those algorithms should be initialized by manually specifying the total number of superpixels, which could hardly suit to various images under diverse environments.

**Figure 1:**
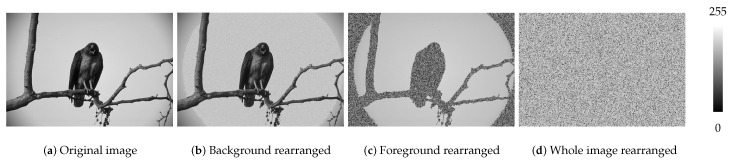
Conventional image entropy is ineffective at responding to pixel patterns over spatial. Although the image may show significantly different patterns, it must derive identical image entropy if they have the same set of pixels. (**a**) The eagle image is selected from BSD300, with a background of sky and the foreground of one eagle standing on a tree. Its pixels at the background, foreground, and the whole regions are rearranged respectively so that generate 3 different images, as shown in (**b**–**d**). Their entropy equals 6.3884 although the image contents are different.

Secondly, conventional image entropy [23] merely involves the overall intensity statistics which cannot respond to intensity patterns over spatial, as shown in Figure 1. Such intensity or visual patterns over spatial are definitely crucial to any biological or artificial vision system. First of all, the ventral and dorsal two visual pathways transmit and ’manipulate’ the physical appearance and spatial information, respectively [24], viz., visual patterns involve the visuospatial domain. Second and more apparently, Gestalt grouping rules involve stimuli patterns of both aspects [25]. Last but not the least, surrounding objects (i.e., visual context) modulate the firing rate of neurons representing the target object [26,27], which affects the visual awareness inherently.

In this paper, we propose a context-aware superpixel (CASP) algorithm and the bilateral entropy (BE), aiming to test the encoding efficiency of Gestalt grouping rules. CASP iteratively aggregates pixels from coarse to fine and each clustering iteration explicitly follows the similarity and proximity grouping rules. The coarse-level superpixels with contextual information guide to generating superpixels in a fine-level. Besides CASP, we propose BE with two aspects, that is, conditional intensity entropy and spatial occupation entropy which involve local image coherence, spatial occupation respectively. With extensive experiment results, CASP demonstrates promising superpixel segmentation performance, compared with SLIC and LSC; while CASP will achieve the smallest entropy of all three criteria when it segments coherent objects out precisely. Besides, further results demonstrate a strong correlation between local image coherence and superpixel segmentation performance, based on Pearson’s correlation coefficient, in a collection of data with a total of 120 samples. Our results support the visual system inclines to encode visual information in the most efficient fashion.

Our paper is organized as follows. In Section 2, we will describe the workflow of CASP. In Section 3, we will give a detailed description of BE. In Section 4, we will demonstrate the effectiveness of CASP with respect to both segmentation performance and encoding efficiency. Finally, in Section 5, we will discuss and conclude our works.

## 2. Context-Aware Superpixel (CASP)

We design CASP to generate a hierarchy of large to small superpixels that represent the input image from coarse to fine. To achieve this, CASP iteratively partitions an image, and each iteration contains 3 stages, including (1) *similarity clustering*, which aggregates pixels with similar physical appearance; and (2) *proximity clustering*, which defines proximity by connectedness and isolates spatially unconnected groups; and (3) *merging stage*, which merges tiny groups into the most similar neighbor.

**Figure 2:**
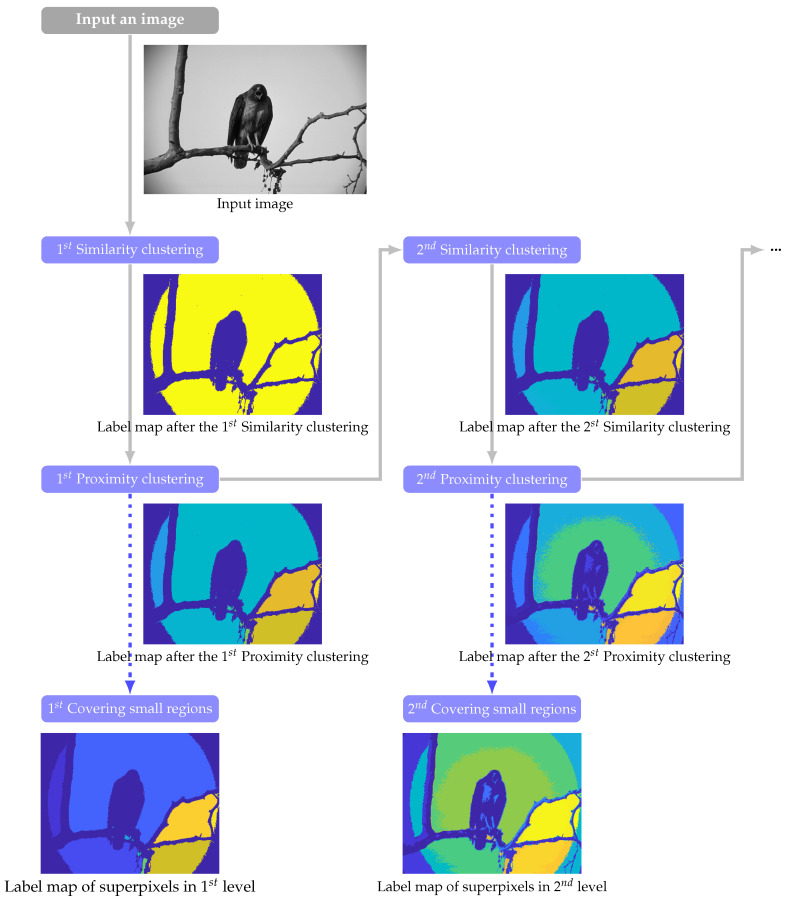
The clustering workflow of context-aware superpixel (CASP). Different colors in label maps denote the adopted cluster labels. We can see the whole yellow background of the outcome of 1^st^ similarity clustering is split into several connected parts by the 1^st^ proximity clustering. The outcome of proximity clustering is not only filled into the next iteration of clustering but also used to generate superpixels.

The workflow of CASP is vividly illustrated in Figure 2, each column, with three blue boxes denoting three clustering stages respectively, demonstrates one clustering iteration; and the image below each box is outcome label map of the corresponding clustering stage. From the left column to the right, CASP generates superpixels from coarse to fine. The preceding clustering iteration generates coarse-level aggregations that serve as the context to subsequent clustering. The coarse-level outcomes are next entered into the next iteration of clustering separately to adopt fine-level ones. By this, CASP can gradually pop out the less salience image regions (i.e., circular halo in the background region), as shown in Figure 2.

In the first stage, CASP employs expectation maximization (EM) clustering in simulating the Gestalt similarity grouping rule. Denote an image with *N* pixels, $I\left(\vec{x}\right)=\left\{I\left({\vec{x}}_{1}\right),\ldots ,I\left({\vec{x}}_{i}\right),\ldots ,I\left({\vec{x}}_{N}\right)\right\}$, EM clustering aggregates them into two coherent groups with labels $\ell \in \left\{\ell_{1},\ell_{2}\right\}$, which intensity distributions are represented by two Gaussian models, with a parameter set $\theta_{t,\;\ell }$ that has maximized the lower-bound of log-likelihood [28],

(1) $$\underset{\theta_{t,\;\ell }^{*}}{\arg\max}\sum_{\ell \in \left\{\ell_{1},\ell_{2}\right\}}p\left(\ell |I\left({\vec{x}}_{i}\right),\theta_{t,\;\ell }\right)\log p\left(I\left({\vec{x}}_{i}\right),\ell |\theta_{t,\;\ell }^{*}\right),$$

where $\theta_{t,\;\ell }=\left\{\mu_{t,\;\ell },\delta_{t,\;\ell }\right\}$ consistes of mean $\mu_{t,\;k}$ and standard deviation (STD) $\delta_{t,\;\ell }$ of the *ℓ* cluster at *t*-th level.

The input pixels are split into two parts, which are denoted by two colors in the label map of 1^st^ similarity clustering, shown in Figure 2. Next, proximity clustering defines connectedness as a type of proximity for simulating Gestalt proximity grouping rule. It detects outcome connectivity of first stage and gives each isolated part a unique label. The label map hence becomes more colorful. After proximity clustering, the adopted clusters may be tiny, even only have 1 pixel. The third stage hereby merges tiny groups into the most similar neighbor. Finally, CASP derives superpixels in a specific coherence level.

Initialized by four parameters. They heuristically adapt the total number of superpixels to diverse images. They will be introduced in Section 4.1 in detail.

## 3. Bilateral Entropy

Gestalt grouping rules do not only shed light on how inner consciousness represents afferent visual stimuli but also are the strategies to aggregate coherent pixels into superpixels. They emphasize the crucial function of both physical appearance and spatial information. However, conventional image entropy cannot respond to intensity patterns over spatial,

(2) $$H_{\mathrm{IM}}\left(I\left(\vec{x}\right)\right)=-\sum_{I(\vec{x})=0}^{255}p\left(I\left(\vec{x}\right)\right)\log_{2}p\left(I\left(\vec{x}\right)\right),$$

where $H_{\mathrm{IM}}\left(I\left(\vec{x}\right)\right)$ merely measures overall intensity statistics. If images share the same intensity histogram, it will generate an identical value, regardless of their content differences.

To evaluate the encoding efficiency of superpixels or image coherence, we propose bilateral entropy that extends the conventional image entropy for concurrently considering the local image coherence and spatial occupation,

(3) $$H_{\mathrm{BE}}\left(I\left(\vec{x}\right),L\left(\vec{x}\right)\right)=-\sum_{I\left(\vec{x}\right)=0,L\left(\vec{x}\right)=1}^{255,K}p\left(I\left(\vec{x}\right),L\left(\vec{x}\right)\right)\log_{2}p\left(I\left(\vec{x}\right),L\left(\vec{x}\right)\right),$$

where the joint probability distribution

(4) $$p\left(I\left(\vec{x}\right),L\left(\vec{x}\right)\right)=p\left(I\left(\vec{x}\right)|L\left(\vec{x}\right)\right)p\left(L(\vec{x})\right),$$

thus, we can derive the bilateral entropy in the form,

(5) $$H_{\mathrm{BE}}\left(I\left(\vec{x}\right),L\left(\vec{x}\right)\right)=-\sum_{I\left(\vec{x}\right)=0,L\left(\vec{x}\right)=1}^{255,K}p\left(I\left(\vec{x}\right)|L\left(\vec{x}\right)\right)p\left(L\left(\vec{x}\right)\right)\log_{2}\left\{p\left(I\left(\vec{x}\right)|L\left(\vec{x}\right)\right)p\left(L\left(\vec{x}\right)\right)\right\},$$

where $L(\vec{x})$ denotes the label of superpixels; $p(L\left(\vec{x}\right))$ is the occupation ratio; and $p(I\left(\vec{x}\right)|L\left(\vec{x}\right))$ is local intensity histogram of $L(\vec{x})$-th superpixel. Typically, $H_{\mathrm{BE}}\left(I\left(\vec{x}\right),L\left(\vec{x}\right)\right)$ can be rewritten by

(6) $$H_{\mathrm{BE}}\left(I\left(\vec{x}\right),L\left(\vec{x}\right)\right)=H_{\mathrm{CON}}\left(I\left(\vec{x}\right)|L\left(\vec{x}\right)\right)+H_{\mathrm{OCC}}\left(L(\vec{x})\right),$$

where $H_{\mathrm{CON}}\left(I\left(\vec{x}\right)|L\left(\vec{x}\right)\right)$ and $H_{\mathrm{OCC}}\left(L(\vec{x})\right)$ measure local intensity statistic and spatial occupation respectively. Both terms involve the complexity of a visual task reflected by similarity and proximity Gestalt grouping rules and we propose $H_{\mathrm{BE}}\left(I\left(\vec{x}\right),L\left(\vec{x}\right)\right)$ which is the sum of both terms to integrally measure both aspects with the same significance weight.

## 4. Results

In this section, we will first introduce the parameter settings of CASP then describe the evaluation criteria; and next, two baseline methods will be introduced for comparison. At last, we will report the experimental results w.r.t. superpixel segmentation performance and encoding efficiency.

### 4.1. Parameter Settings

In all experiments, we initialize the parameters of CASP as follows:

1. **Maximal clustering iteration limit $D_{p}$:**$D_{p}$ determines the maximal depth of the superpixel hierarchy, which is 7 to acquire superpixels in 6 levels.
2. **Maximal cluster size ${\mathrm{Mx}}_{c}$:** We set $Mx_{c}$ to 100. During each similarity clustering stage, only the group of input pixels which size exceeds 100 is divided into subgroups.
3. **Minimal cluster size ${\mathrm{Mn}}_{c}$:** We set $Mx_{c}$ to 4. During each merging stage, a cluster with a size smaller than 4 is merged into the most similar neighbor.
4. **neighbourhood range $N_{e}$:** We initialized $N_{e}$ to 8 to retrieve 8 neighbors of a pixel.

### 4.2. Evaluation Criteria

Under-segmentation error (USE), boundary recall (BR) and achievable segmatiaon accuracy (ASA) are the most commonly used criteria to evaluate superpixel segmentation performance. The USE, ASA and BR close to 0, 1 and 1 respectively are desired. Denote a ground-truth segmentation of an image as $G=\left\{g_{1},\ldots ,g_{i},\ldots ,g_{K^{\prime }}\right\}$ and a superpixel partition as $S=\left\{s_{1},\ldots ,s_{j},\ldots ,s_{K}\right\}$.

1. USE: Given a ground-truth segmentation $g_{i}$, USE measures the fraction of pixels that leak across its boundaries caused by the overlapping superpixels $s_{j}$ [29],
  (7) $$USE=\frac{\sum_{s_{j}\in S:Area\left(s_{j}\cap g_{i}\right)>B_{j}}Area\left(s_{j}\right)-Area\left(g_{i}\right)}{Area(g_{i})},$$
  where Area(·) counts the pixel amount of an image region. We set $B_{j}$ to 25 percent of $Area(s_{j})$ because CASP may generate the smallest superpixel with a size of 4. We use the average of all segments in G.
2. BR: Given the boundaries of ground-truth segmentations, denoted by δG, BR measures the percentage of the ground-truth boundaries recovered by superpixels, denoted by δS. We compute BR by
  (8) $$BR=\frac{\sum_{p\in \delta G}l\;l\left(\min_{q\in \delta S}∥p-q∥<\epsilon \right)}{\left|\delta G\right|},$$
  where *p* and *q* denote the location of pixels belong to δG and δS respectively. We set ϵ to 1 so that count a pixel at *q* that falls within a 1 pixel range of *q*, which operation is denoted by ll.
3. ASA: Given all ground-truth segmentations G, ASA gives the largest overlapping area between superpixels and the ground truth segments [30]
  (9) $$ASA=\frac{\sum_{s_{j}\in S}\max_{\theta_{\left(g_{i}\in G\right)}}\left\{Area\left(s_{j}\right)\cap Area\left(g_{i}\right)\right)\}}{\sum_{g_{i}\in G}Area\left(g_{i}\right)}.$$
  In general, ASA is the highest achievable accuracy for object segmentation that utilizes superpixels as units.

### 4.3. Baseline Methods

We compare CASP with two baseline methods, SLIC and LSC, which need to be initialized by the total number of superpixels. Because CASP heuristically generates the total numbers in 6 levels, we specify them to the baseline methods, which will be listed in Section 4.4.

1. **SLIC** employs a balance factor to weigh the importance of color and coordinates [21]. We initialize the balance factor 40 and merged tiny image regions that radius is smaller than 1.
2. **LSC** approximates the coherence metric using a kernel function and maps color and coordinates to a high dimensional feature space [22]. We set the compactness factor to 0.066.

### 4.4. Evaluation Datasets

**Figure 3:**
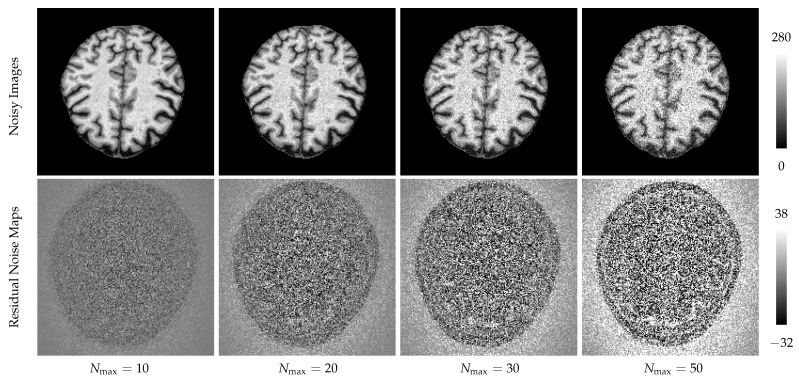
After added Rician noise to the ground truth data, we can see in the first row that the image is corrupted by the noise in different levels, denoted by $N_{\max}$. After subtracting the noisy image from the ground truth, we derived residual maps of Rician noise shown in the second row. Because the Rician noise is signal dependent, we can see apparent content textures in the residual noise maps.

**Figure 4:**
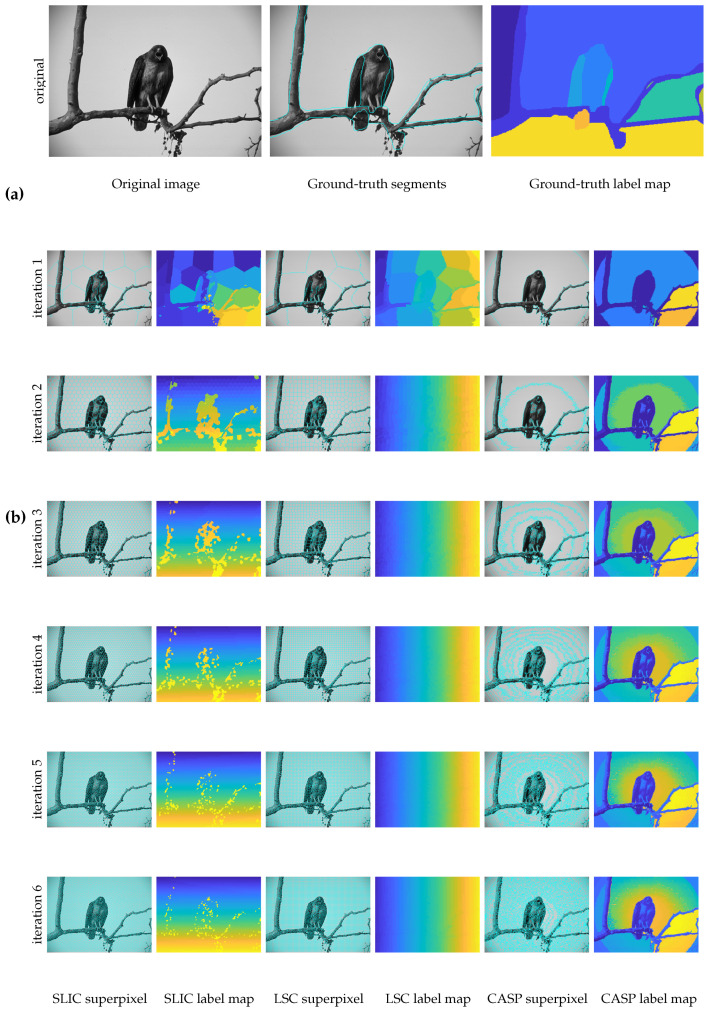
Coarse to fine superpixels on the eagle image. Owing to the hierarchically clustering, CASP can even capture halo in a circular pattern which is neither a salient feature of human vision nor of baseline methods. (**a**) The ground truth segmentation of the eagle image; (**b**) superpixel segmentation results of three methods in 6 levels.

1. **BSD300** [31] is a natural image database, which suits conventional methods working in chrominance space. However, the labels are manually placed, which are not precise, shown in Figure 4a. We hence do not use it for quantitative evaluation. To initialize LSC and SLIC, the superpixel numbers in 6 levels are [24,532,1121,2253,4390,6115].
2. **MRBrainS** [32] contains 3T T1-weighted magnetic resonance (MR) brain scans. We employ 4 subjects from the training dataset, each of which contains 48 slices. The labels are carefully placed, including gray matter (GM), white matter (WM) and cerebrospinal fluid (CSF). The background image regions are removed out and filled with an intensity of 0. As the MR images are in the UINT16 encoding format, which cannot be used by the LSC, we transform the intensity range to UINT8 and duplicate each slice to 3-channels. To initialize LSC and SLIC, the superpixels number of 6 levels are [2,15,136,392,595,615].
  And next, for testing the segmentation performance under noise corruption, we add spatially varying Rician noise to MR magnitudes. To achieve this, we firstly simulate phase maps according to the literature [33]; and next, we generated both real and imaginary components and added spatially varying Gaussian noise to each component separately. The noise level follows the 2D Gaussian distribution in the same manner as the literature [34]. Based on the noisy components, we eventually generate the noisy magnitude with Rician noise using the simple sum of square (SoS) image reconstruction manner [35]. More specifically, we added 4 levels of spatially varying Gaussian noise to the complex components, with maximal noise levels $N_{\max}=\left\{10,20,30,50\right\}$. The noisy images and residual noise maps are shown in Figure 3.

### 4.5. Segmentation Performance

Figure 4a shows the ground-truth segments of the eagle image, including the superpixel boundaries and a label map for clear graphic-illustration. We can find ground-truth segments do not well adhere to true boundaries. We hence do not quantitatively evaluate superpixels on BSD300. Our qualitative results are shown in Figure 4b. Prominently, we can find CASP partitioned the image from coarse to fine, image details are gradually popped out.

**Figure 5:**
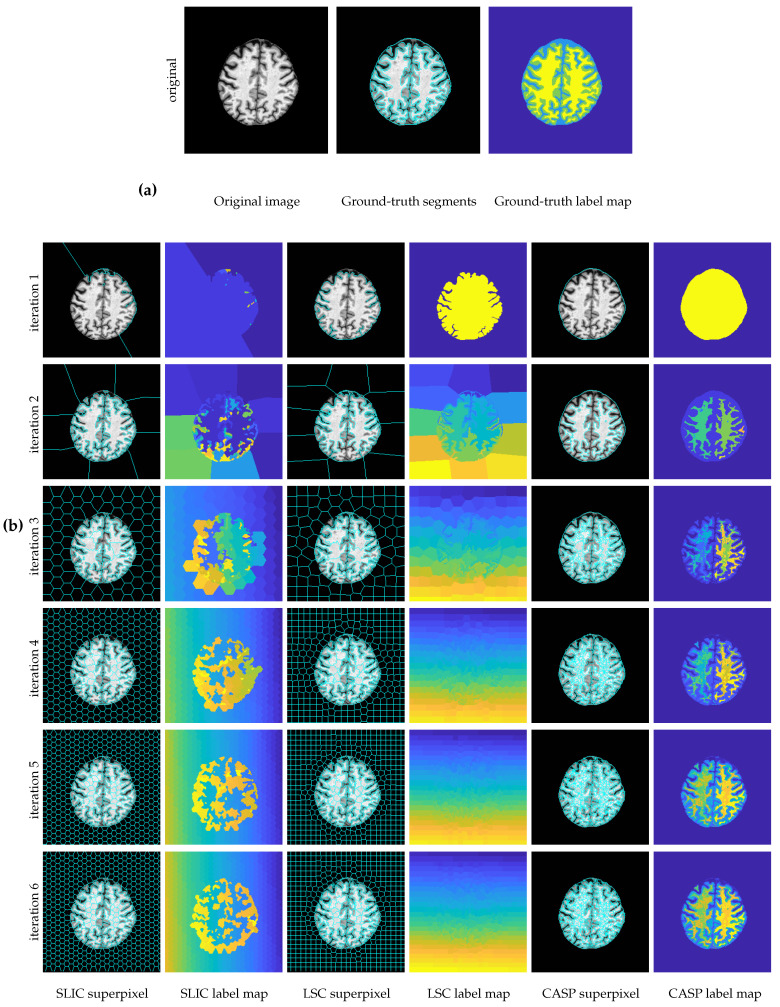
Coarse to fine superpixels on the magnetic resonance (MR) brain image. Due to manually specifying the total number of superpixels, simple linear iterative clustering (SLIC) and linear spectral clustering (LSC) evenly generate superpixels in the constant background image region. By contrast, CASP only generates one superpixel for representing background, indicating high-level adaptivity. (**a**) The ground truth segmentation of a MR slice; (**b**) superpixel segmentation results of three methods in 6 levels.

Figure 5a shows an MR brain slice from MRBrainS. The ground truth segments with 4 labels are denoted by 4 different colors in the label map. We can see from Figure 5b, in the row of iteration 1, SLIC fails to segment the intracranial region, while LSC and CASP successfully achieve this. More apparently, CASP aggregates the background as one unit and extracts more details in the intracranial regions.

**Figure 6:**
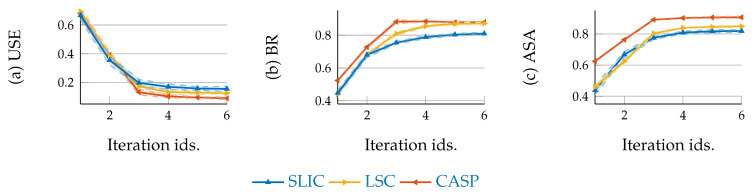
CASP achieves better segmentation performance than baseline methods, with respect to under-segmentation error (**a**), boundary recall (**b**), and (**c**) achievable segmentation accuracy.

Figure 6a–c demonstrate the quantitative superpixel segmentation performance w.r.t. USE, BR, and ASA respectively. The solid curves denote the mean across average values of 4 subjects, while the shades denote the STD. We can find the CASP outperforms others.

### 4.6. Encoding Efficiency

**Figure 7:**
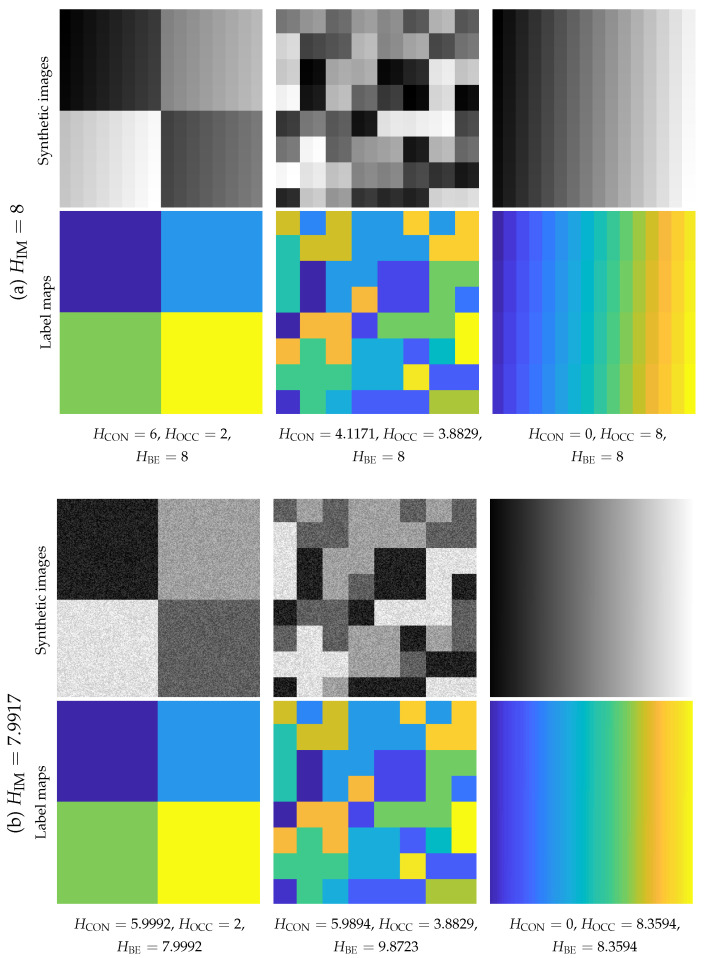
Entropy values of synthetic images. Denote a superpixel as one object. The complementary role of $H_{\mathrm{CON}}$ and $H_{\mathrm{OCC}}$ indicates that uncertainty is transferred from that within objects to that among objects. (**a**) $H_{\mathrm{BE}}$ degenerates to $H_{\mathrm{IM}}$ when $H_{\mathrm{CON}}=0$ and $H_{\mathrm{OCC}}=H_{\mathrm{IM}}$. (**b**) The increasing object amount elevates $H_{\mathrm{BE}}$, while we can find a proper arrangement of pixels may decrease the $H_{\mathrm{BE}}$.

We first synthesize two groups of toy images as shown in Figure 7a,b respectively. Each group contains three images with the same pixel set but in 3 different patterns. From the left column to the right, the superpixel number increases significantly.

In Figure 7a, each of the three synthetic images only has 256 pixels, with a size of 16×16. Every pixel is unique in intensity, which value involves the UINT8 range from 0 to 255. The synthetic images along with label maps were used to generate the $H_{\mathrm{OCC}}\left(L(\vec{x})\right)$, $H_{\mathrm{CON}}\left(I\left(\vec{x}\right)|L\left(\vec{x}\right)\right)$, and $H_{\mathrm{BE}}\left(I\left(\vec{x}\right),L\left(\vec{x}\right)\right)$, attached below each label map. We can find $H_{\mathrm{BE}}\left(I\left(\vec{x}\right),L\left(\vec{x}\right)\right)$ degenerates to $H_{\mathrm{IM}}\left(I\left(\vec{x}\right)\right)$, which equals to 8 all the times. From the left column to the right, with the increase of superpixel number, $H_{\mathrm{OCC}}\left(L(\vec{x})\right)$ increases, and $H_{\mathrm{CON}}\left(I\left(\vec{x}\right)|L\left(\vec{x}\right)\right)$ decreases synchronously, demonstrating their complementary role. When $H_{\mathrm{CON}}\left(I\left(\vec{x}\right)|L\left(\vec{x}\right)\right)=0$, $H_{\mathrm{BE}}\left(I\left(\vec{x}\right),L\left(\vec{x}\right)\right)$ degenerates to $H_{\mathrm{OCC}}\left(L(\vec{x})\right)$. The degeneration indicates that the uncertainty contribution will only exist in the group of noticed objects if a visual system has focused on the constant objects. Typically, such uncertainty among objects may equal to the one in their exterior physical appearance.

In Figure 7b, the synthetic images had a size of 256×256. Pixels are roughly in 4 intensity levels, each of which is biased by small-level Gaussian noise. These pixel intensities may overlap. In the third column, we organize the image so that the same pixels belong to one superpixel. The intensity of different superpixels may be the same. We can find the bilateral entropy decreases with the increase of local coherence. Typically, it is interesting to find even when the total number of superpixels is far more than the one in the middle column, and the bilateral entropy has been decreased.

**Figure 8:**
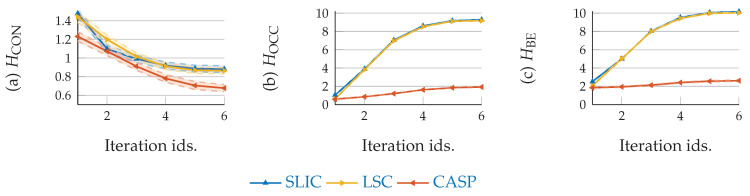
CASP derives less entropy values than based line methods. (**a**) CASP generates superpixels with less $H_{CON}$, indicating a higher level of local intensity coherence; (**b**) Also, $H_{OCC}$ derived from CASP is smaller than other baseline methods; (**c**) Accordingly, $H_{BE}$ derived from CAPS is the smallest.

The curves and shades in Figure 8a–c demonstrate the mean and STD values across 4 subjects of $H_{\mathrm{CON}}\left(I\left(\vec{x}\right)|L\left(\vec{x}\right)\right)$, $H_{\mathrm{OCC}}\left(L(\vec{x})\right)$, and $H_{\mathrm{BE}}\left(I\left(\vec{x}\right),L\left(\vec{x}\right)\right)$, respectively. The descending curve slope in Figure 8a indicates the enhancement of local coherence within superpixels. While as shown in Figure 8b, the increasing amount of superpixels drives ascending curve slope. The integral function of both terms is illustrated by $H_{\mathrm{BE}}\left(I\left(\vec{x}\right),L\left(\vec{x}\right)\right)$, shown in Figure 8c. Most prominently, we can see CASP achieves the smallest values for all three cases. In addition, CASP increases $H_{\mathrm{OCC}}$ and $H_{\mathrm{BE}}$ much less, outperforming LSC and SLIC significantly, although the total number of superpixels of the three methods are roughly identical.

### 4.7. Segmentation Robustness and Local Image Coherence

Since the human visual system is widely believed to be robust to arbitrary interference, we further evaluate CASP on the MR data, which are manually added spatially varying Rician noise. Accordingly, we have a large number of MR data, both noise free and noisy ones. Ultimately, we evaluate the correlation between local coherence, $H_{\mathrm{CON}}\left(I\left(\vec{x}\right)|L\left(\vec{x}\right)\right)$ and segmentation performance, including USE, BR and ASA, respectively.

**Figure 9:**
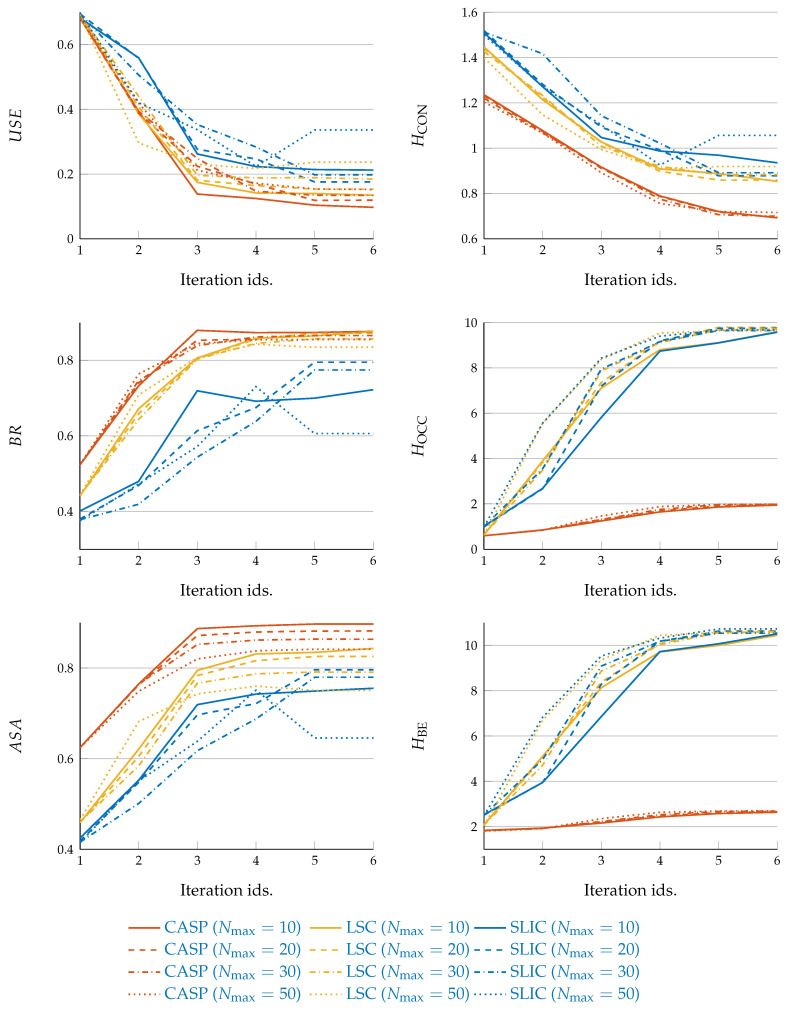
Under image corruption that is caused by no matter slight or severe noise, CASP demonstrates better performance than baseline methods. (Denote noise level $N_{\max}$)

Figure 9 shows both superpixel segmentation, which is in the left column, and encoding efficiency performance, which is in the right column. Different methods are marked by curve colors, while the performance under different noise conditions are denoted by the line types. In general, the higher the noise level, the worse the segmentation performance, and the robustness of a method behaves through, besides better performance, the shape consistency of the group of curves demonstrating under different noise levels. We can find in Figure 9 that, CASP demonstrates superiority on the two aspects, which curves not only locate at places indicating better performance but also demonstrate higher-level consistency in curve shape. By contrast, the curves of SLIC demonstrate arbitrary shapes indicating vulnerability to noise.

Comparing the two sub-figures in the first row of Figure 9, it is interesting to find the $H_{\mathrm{CON}}$ curves of SLIC seem to correlate with its USE curves. To find out the relationship between local image coherence and superpixel segmentation performance, we employ Pearson’s correlation coefficient (PCC).

**Figure 10:**
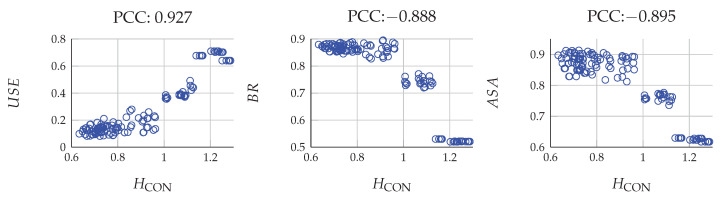
Pearson’s correlation coefficient between local image coherence, $H_{\mathrm{CON}}$ and superpixel segmentation performance w.r.t. USE, BR, and ASA. USE has a positive correlation with $H_{\mathrm{CON}}$, while BR and ASA negatively correlate with $H_{\mathrm{CON}}$.

Figure 10 illustrate the correlation between $H_{\mathrm{CON}}$ and USE, BR, and ASA respectively. Each blue circle denotes a subject (4 in total) that is represented by superpixels in a specific iteration (6 in total) and noise level (5 in total, including noise free). There are 120 samples used to generate PCC in total. No matter from the shape of scattered circles or PCC values, our results support that local image coherence and superpixel segmentation has a strong correlation.

## 5. Discussions and Conclusions

In this paper, we proposed a superpixel clustering algorithm, named context-aware superpixel, which aggregated pixels explicitly following two of Gestalt grouping rules and top-down hierarchical principle. Next, we proposed the conditional intensity entropy $H_{\mathrm{CON}}\left(I\left(\vec{x}\right)|L\left(\vec{x}\right)\right)$, spatial occupation entropy $H_{\mathrm{OCC}}\left(L(\vec{x})\right)$, and bilateral entropy $H_{\mathrm{BE}}\left(I\left(\vec{x}\right),L\left(\vec{x}\right)\right)$ in the context of superpixel segmentation to test the encoding efficiency of local image coherence, as an alternative of the complex brain.

In the superpixel segmentation task, as shown in Figure 4 and Figure 5, CASP generated superpixels that fitted image contents more precisely than SLIC and LSC. The superiority of CASP attributed to the hierarchical image representation. First, CASP avoided to manually specify the total number of superpixels, like the hierarchical model enables the learning of prior knowledge [36,37]; and second, CASP discerned fine details based on a coarse aggregation, which took advantage of the information in coarse-level [26,38]. As a result, the superpixels adopted by CASP in the fine-level reserved the boundaries of superpixels in the coarse-level. By contrast, the well-segmented boundaries of SLIC and LSC in coarse-level did not present in the fine-level. They can not generate the hierarchical representations although they were initialized by a list of small to large numbers of superpixels. As shown in Figure 6 and Figure 9, quantitative results consistently supported the superiority of CASP.

Theoretically speaking, superpixel methods simulate how the human visual system reduces redundancy. Luck and Vogel (1997) claims that what maintains in visual working memory are the integrated objects rather than individual features [39]; besides, the working memory is only capable of maintaining a limited number of objects at the same time [40]. Therefore, discarding trivial information that does not affect recognition improves the visual efficiency [41].

Meanwhile, Gestalt perceptual rules play a crucial role in both visual working memory and superpixel methods, and they manifest the visual efficiency of the human visual system. Most prominently, contrasting to Barlow’s (1961) redundancy reduction [41], Gestalt grouping rules, or superpixels that did not discard any information but did structure pixels, demonstrated improvement on encoding efficiency. To test the encoding efficiency of superpixels, we proposed three entropy criteria. From Equation (6), we can find the $H_{\mathrm{CON}}\left(I\left(\vec{x}\right)|L\left(\vec{x}\right)\right)$ and $H_{\mathrm{OCC}}$ were the two aspects of $H_{\mathrm{BE}}$ that concurrently measured local image coherence and spatial occupation. From Figure 8 and Figure 9, we can see CASP achieved less entropy than LSC and SLIC in all three criteria. Specifically, the local image coherence strongly correlates with superpixel segmentation performance, as supported by Pearson’s correlation analysis shown in Figure 10.

To sum up, partitioning image according to image coherence, although without any loss of detail information, induced less entropy. Such results support Gestalt minimal principle and might imply an underlying principle, that is, the minimum entropy principle that a visual system reduces the uncertainty existing in visual stimuli efficiently if the stimuli have a minimal entropy.

Compared to Edwin Jaynes’ (1957) maximum entropy principle, our conclusion seems to contradict. We denote different two aspects. For Edwin Jaynes’, the probability distribution with maximum entropy is the a priori criterion used in an inference process when little or even no information is available [42]. During the inference iterations, this prior distribution changes to have a sharp shape with newly available information [43]. By contrast, for ours, the minimum entropy principle involved the statistics within the data, and it was a measure of uncertainty of the image itself, with reference to local image coherence and spatially occupation. Although they were both named entropy, their roles are different.

More specifically, it is worth noting that the proposed entropy criteria involve the image structure in a hierarchy captured by CASP following the top-down manner. If considering such a hierarchy as the inner structure of visual awareness, each superpixel may involve one visual object. Accordingly, the hierarchical structure is the information that is transmitted from raw stimuli to the visual system. Denote the mutual information

(10) $$I_{mu}\left(I\left(\vec{x}\right),L\left(\vec{x}\right)\right)=H_{\mathrm{IM}}\left(I(\vec{x})\right)-H_{\mathrm{CON}}\left(I\left(\vec{x}\right)|L\left(\vec{x}\right)\right),$$

since $H_{\mathrm{IM}}\left(I(\vec{x})\right)$ must be a constant, $I_{mu}\left(I\left(\vec{x}\right),L\left(\vec{x}\right)\right)$ is determined by the $H_{\mathrm{CON}}\left(I\left(\vec{x}\right)|L\left(\vec{x}\right)\right)$. Thus, maximizing $I_{mu}\left(I\left(\vec{x}\right),L\left(\vec{x}\right)\right)$ equals to minimizing $H_{\mathrm{CON}}\left(I\left(\vec{x}\right)|L\left(\vec{x}\right)\right)$. Because a smaller $H_{\mathrm{CON}}\left(I\left(\vec{x}\right)|L\left(\vec{x}\right)\right)$ indicates a higher-level of intensity coherence of a superpixel, we can summarize that the way to represent image coherence minimizes the conditional entropy $H_{\mathrm{CON}}\left(I\left(\vec{x}\right)|L\left(\vec{x}\right)\right)$ and maximizes mutual information $I_{mu}\left(I\left(\vec{x}\right),L\left(\vec{x}\right)\right)$, maximizes the information transmission, indicating visual efficiency. On the other side, the statistics of local image coherence reflect the complexity within the data. Thus, the local image coherence that is reflected by $H_{\mathrm{CON}}\left(I\left(\vec{x}\right)|L\left(\vec{x}\right)\right)$ can be used to depict the complexity within the data, and it is obvious that an image with a simple structure is easily be recognized by human and be represented by CASP. We, therefore, suggest that the coherence-based image structure might be one type of the data structure of unlabelled data, anticipated by Hinton (2018) [44], which can be fully exploited. Furthermore, it is the coherence rather than the individual features that improve the encoding efficiency, and our results may respond to the query whether feature invariance is always an optimal strategy for biological and artificial vision [38].

As far as we know, this work is the first investigation to test Gestalt minimum hypothesis utilizing a superpixel algorithm (i.e., CASP) and entropy criteria (i.e., $H_{\mathrm{CON}}\left(I\left(\vec{x}\right)|L\left(\vec{x}\right)\right)$, $H_{\mathrm{OCC}}\left(L(\vec{x})\right)$ and $H_{\mathrm{CON}}\left(I\left(\vec{x}\right)|L\left(\vec{x}\right)\right)$). However, there are limitations worth to note. First, the artificial vision system is too simple to understand the true underlying visual mechanisms, and typically, the adopted entropy value might is unable to indicate the true number of firing neurons. Second, the bilateral entropy can only weigh the encoding efficiency of patterns at the very early perceptual stage, viz., the image coherence. Even so, the strong correlation between local image coherence and superpixel segmentation performance suggests the potential to exploit the proposed entropy criteria as constraints in designing a loss function of the segmentation tasks. Besides the intensity coherence over spatial, as claimed by Attneave (1956), visual patterns also demonstrate many other types, such as shape, angle, curvature, and so forth [13]. Simultaneously, Gestalt Berlin school claims a whole-part relationship that the whole is different from the sum of the parts [2]. Both reasons let us know that the proposed entropy criteria may fail to respond to more complex patterns. Therefore, we suggest that it should design specific entropy criterion to measure the encoding efficiency of complex patterns corresponding to specific vision level, by which human vision and computer vision researchers could test and refine their hypotheses theoretically and quantitatively.

## Abbreviations

The following abbreviations are used in this manuscript:

CASP	Context-Aware SuperPixel
SLIC	Simple Linear Iterative Clustering
LSC	Linear Spectral Clustering
USE	Under-Segmentation Error
BR	Boundary Recall
ASA	Achievable Segmatiaon Accuracy
MR	Magnetic Resonance
STD	Standard Deviation
SoS	Sum of Square
PCC	Pearson’s Correlation Coefficient

## References

[1] Shi J. (2000). Normalized cuts and image segmentation. IEEE Trans. Pattern Anal. Mach. Intell..

[2] Wagemans J., Elder J.H., Kubovy M., Palmer S.E., Peterson M.A., Singh M., von der Heydt R. (2012). A century of Gestalt psychology in visual perception: I. Perceptual grouping and figure–ground organization. Psychol. Bull..

[3] Liu Y.J., Yu M., Li B.J., He Y. (2017). Intrinsic manifold SLIC: A simple and efficient method for computing content-sensitive superpixels. IEEE Trans. Pattern Anal. Mach. Intell..

[4] Liu F., Feng J., Su W., Lv Z., Xiao F., Qiu S. Normalized Euclidean Super-Pixels for Medical Image Segmentation. Proceedings of the International Conference on Intelligent Computing.

[5] Huo J., Wu J., Cao J., Wang G. (2018). Supervoxel based method for multi-atlas segmentation of brain MR images. NeuroImage.

[6] Kaufhold J., Collins R., Hoogs A., Rondot P. Recognition and segmentation of scene content using region-based classification. Proceedings of the IEEE International Conference on Pattern Recognition (ICPR).

[7] Fulkerson B., Vedaldi A., Soatto S. Class segmentation and object localization with superpixel neighborhoods. Proceedings of the IEEE International Conference on Computer Vision (ICCV).

[8] Wang S., Lu H., Yang F., Yang M.H. Superpixel tracking. Proceedings of the IEEE International Conference on Computer Vision (ICCV).

[9] Gao T., Gao Z., Li J., Sun Z., Shen M. (2011). The perceptual root of object-based storage: An interactive model of perception and visual working memory. J. Exp. Psychol. Hum. Percept. Perform..

[10] Peterson D.J., Berryhill M.E. (2013). The Gestalt principle of similarity benefits visual working memory. Psychon. Bull. Rev..

[11] Gao Z., Gao Q., Tang N., Shui R., Shen M. (2016). Organization principles in visual working memory: Evidence from sequential stimulus display. Cognition.

[12] Attneave F. (1954). Some informational aspects of visual perception. Psychol. Rev..

[13] Attneave F., Arnoult M.D. (1956). The quantitative study of shape and pattern perception. Psychol. Bull..

[14] Marblestone A.H., Wayne G., Kording K.P. (2016). Toward an integration of deep learning and neuroscience. Front. Comput. Neurosci..

[15] Rajalingham R., Issa E.B., Bashivan P., Kar K., Schmidt K., DiCarlo J.J. (2018). Large-scale, high-resolution comparison of the core visual object recognition behavior of humans, monkeys, and state-of-the-art deep artificial neural networks. J. Neurosci..

[16] Gangopadhyay P., Das J. (2019). Do Primates and Deep Artificial Neural Networks Perform Object Categorization in a Similar Manner?. J. Neurosci..

[17] Bronstein M.M., Bruna J., LeCun Y., Szlam A., Vandergheynst P. (2017). Geometric deep learning: Going beyond euclidean data. IEEE Signal Process. Mag..

[18] Wang H., Feng J., Bu Q., Liu F., Zhang M., Ren Y., Lv Y. (2018). Breast mass detection in digital mammogram based on Gestalt psychology. J. Healthc. Eng..

[19] Wang H., Feng J., Zhang Z., Su H., Cui L., He H., Liu L. (2018). Breast mass classification via deeply integrating the contextual information from multi-view data. Pattern Recognit..

[20] Liu F., Feng J., Chen G., Wu Y., Hong Y., Yap P.T., Shen D. (2019). DeepBundle: Fiber Bundle Parcellation with Graph Convolution Neural Networks. arXiv.

[21] Achanta R., Shaji A., Smith K., Lucchi A., Fua P., Süsstrunk S. (2012). SLIC superpixels compared to state-of-the-art superpixel methods. IEEE Trans. Pattern Anal. Mach. Intell..

[22] Li Z., Chen J. Superpixel segmentation using linear spectral clustering. Proceedings of the IEEE Conference on Computer Vision and Pattern Recognition (CVPR).

[23] González R.C., Woods R.E., Eddins S.L. (2009). Digital Image Processing Using MALAB.

[24] Ungerleider L.G., Haxby J.V. (1994). ‘What’and ‘where’in the human brain. Curr. Opin. Neurobiol..

[25] Koffka K. (1922). Perception: An introduction to the Gestalt-Theorie. Psychol. Bull..

[26] Bar M. (2004). Visual objects in context. Nat. Rev. Neurosci..

[27] Lisman J. (2015). The challenge of understanding the brain: Where we stand in 2015. Neuron.

[28] Do C.B., Batzoglou S. (2008). What is the expectation maximization algorithm?. Nat. Biotechnol..

[29] Levinshtein A., Stere A., Kutulakos K.N., Fleet D.J., Dickinson S.J., Siddiqi K. (2009). Turbopixels: Fast superpixels using geometric flows. IEEE Trans. Pattern Anal. Mach. Intell..

[30] Liu M.Y., Tuzel O., Ramalingam S., Chellappa R. Entropy rate superpixel segmentation. Proceedings of the IEEE Conference on Computer Vision and Pattern Recognition (CVPR).

[31] Martin D., Fowlkes C., Tal D., Malik J. A Database of Human Segmented Natural Images and its Application to Evaluating Segmentation Algorithms and Measuring Ecological Statistics. Proceedings of the IEEE International Conference on Computer Vision (ICCV).

[32] Mendrik A.M., Vincken K.L., Kuijf H.J., Breeuwer M., Bouvy W.H., De Bresser J., Alansary A., De Bruijne M., Carass A., El-Baz A. (2015). MRBrainS challenge: Online evaluation framework for brain image segmentation in 3T MRI scans. Comput. Intell. Neurosci..

[33] Pizzolato M., Fick R., Boutelier T., Deriche R. (2016). Noise floor removal via phase correction of complex diffusion-weighted images: Influence on DTI and Q-space metrics. International Conference on Medical Image Computing and Computer-Assisted Intervention.

[34] Liu F., Feng J., Yap P.T., Shen D. (2019). Multi-Kernel Filtering: An Extension of Bilateral Filtering Using Image Context. arXiv.

[35] Chen G., Dong B., Zhang Y., Lin W., Shen D., Yap P.T. (2019). Denoising of Infant Diffusion MRI Data via Graph Framelet Matching in *x*-*q* Space. IEEE Trans. Med. Imaging.

[36] Friston K. (2003). Learning and inference in the brain. Neural Netw..

[37] Badcock P.B., Friston K.J., Ramstead M.J., Ploeger A., Hohwy J. (2019). The hierarchically mechanistic mind: An evolutionary systems theory of the human brain, cognition, and behavior. Cogn. Affect. Behav. Neurosci..

[38] Lu Y., Yin J., Chen Z., Gong H., Liu Y., Qian L., Li X., Liu R., Andolina I.M., Wang W. (2018). Revealing detail along the visual hierarchy: Neural clustering preserves acuity from V1 to V4. Neuron.

[39] Luck S.J., Vogel E.K. (1997). The capacity of visual working memory for features and conjunctions. Nature.

[40] Baddeley A. (1992). Working memory. Science.

[41] Barlow H.B. (1961). Possible principles underlying the transformation of sensory messages. Sens. Commun..

[42] Jaynes E.T. (1957). Information theory and statistical mechanics. Phys. Rev..

[43] Jaynes E.T. (1957). Information theory and statistical mechanics. II. Phys. Rev..

[44] Hinton G. (2018). Deep learning—A technology with the potential to transform health care. J. Am. Med. Assoc..

